# A Retrospective Analysis of the Clinical and Radiological Profile of Patients Admitted With Emphysematous Pyelonephritis

**DOI:** 10.7759/cureus.80893

**Published:** 2025-03-20

**Authors:** Joy Kumar, Nanda Krishna, Abhishek Goyal, Udeept Sindhu

**Affiliations:** 1 Internal Medicine, Kasturba Medical College, Manipal Academy of Higher Education (MAHE), Manipal, IND

**Keywords:** dj stenting, emphysematous pyelonephritis, esbl, escherichia coli, mortality, outcome, prognosis, pyelonephritis, risk factors, urinary tract infection

## Abstract

Background: Emphysematous pyelonephritis (EPN) is a potentially fatal infection of the kidney, predominantly affecting diabetic patients. Despite advances in management, predictors of mortality remain incompletely understood. Analysing the factors associated with a high risk of death can help in identifying high-risk EPN patients and initiating early, aggressive therapy. This study was conducted with the aim of understanding and describing the clinical, biochemical, and microbiological profile of patients diagnosed with EPN and analysing the factors influencing mortality.

Methods: A retrospective observational study was conducted at Kasturba Hospital, Manipal (2017-2022). Data from 117 EPN patients were collected and analysed for demographic, clinical, laboratory, microbiological, and radiological characteristics. Primary endpoints used were successful treatment and all-cause mortality to assess markers of mortality. Risk factors for mortality were assessed using independent samples t-tests. Statistical significance was set at p<0.05.

Results: A total of 117 patients were included in our study. The mean age was 55.5 years, with a female predominance (56.4%). The most common symptom reported was flank pain (77.8%). Diabetes mellitus (DM) was the most common risk factor identified in 96.6% of patients, and Escherichia coli (E. coli) was the most common isolated organism (54.7%). Most patients had Class 1 (33.3%) and Class 2 (28.2%) disease. The average duration of inpatient treatment was reported to be 17.25 days in our patients. Treatment included antibiotics (94%) and drainage procedures, including DJ stenting (55%). Of the 117 patients, mortality occurred in seven patients (6%). Elevated serum creatinine at admission was significantly associated with mortality (p=0.004), while other laboratory markers (hemoglobin A1c (HbA1c), total leukocyte count (TLC), platelet count, sodium) did not show a significant correlation.

Conclusion: Serum creatinine at admission emerged as a key predictor of mortality in EPN, emphasizing the need for early renal function assessment and close monitoring in high-risk patients. The low mortality rate observed in our cohort suggests that early intervention combining empirical antibiotic therapy guided by resistance patterns and minimally invasive drainage techniques may improve survival. Future prospective studies are needed to validate these findings and refine EPN management strategies.

## Introduction

Emphysematous pyelonephritis (EPN) is a life-threatening, necrotising renal infection characterised by gas formation within the renal parenchyma, collecting system, or perinephric space [1,2]. It is predominantly associated with diabetes mellitus (DM) and urinary tract obstructions, with mortality rates historically reported between 40% and 90% [3]. Although the disease remains relatively rare, its aggressive course and potential for severe sepsis, multi-organ failure, and mortality make early diagnosis and intervention critical [4].

The pathogenesis of EPN involves the formation of gas in the necrotic tissue secondary to an infection [2]. E. coli is the most common causative organism, but other bacterial and sometimes fungal infections have also been reported [3]. Bacteria use glucose as a substrate to produce hydrogen and carbon dioxide [3]. Elevated glucose levels and inadequate tissue perfusion and/or oxygenation secondary to microvascular pathologies contribute to the progression of the infection in EPN [3]. Hyperglycemia in diabetes plays a pivotal role in the disease process, providing an ideal substrate for bacterial metabolism while impairing neutrophil function and renal tissue perfusion [3].

Diagnosing EPN can be challenging due to its non-specific symptoms, which often mimic uncomplicated acute pyelonephritis [3,4]. Fever, flank pain, and dysuria are common presentations, but in severe cases, the disease progresses rapidly to septic shock and acute kidney injury (AKI) [3,4]. Radiological assessments with CT scans play a pivotal role in confirming the diagnosis [3]. They also help in identifying urinary tract obstructions, which predispose patients to infection [3].

Management strategies have evolved from aggressive and invasive surgical procedures to more conservative approaches consisting of percutaneous drainage and medications [5,6]. Current evidence suggests initial management involving aggressive resuscitation, drainage, and initiation of appropriate antibiotics following the diagnosis [3]. CT imaging aids in staging EPN severity by using the Huang and Tseng classification [7]. Selected cases of progressive EPN (Class III and IV) with failure to respond to medical management and drainage require a more invasive approach with nephrectomy [3]. Moreover, antibiotic selection remains a challenge due to the increasing prevalence of extended-spectrum beta-lactamase (ESBL) producing organisms, leading to carbapenems emerging as a preferred empirical choice in many centers [3].

Despite advancements in diagnosis and treatment, mortality remains significant, and predictors of poor prognosis are not well-defined [7]. Thrombocytopenia, serum creatinine, and poor blood glucose control are some of the factors that have been previously associated with poor prognosis, necessitating early identification and intervention [7]. Similarly, hypoalbuminemia, shock, polymicrobial infections, and the need for hemodialysis have been shown to be potential indicators of poor outcomes in EPN [5]. However, the degree of their prognostic relevance varies across different studies, warranting further investigation [5,7].

Thus, a detailed revised description of disease characteristics, prognostic indicators, and treatment outcomes is necessary to improve patient stratification and management. This study aims to comprehensively evaluate the clinical, biochemical, and microbiological profile of patients diagnosed with EPN and identify factors influencing mortality. By analysing key laboratory parameters, microbiological isolates, and radiological classification, we seek to determine significant prognostic indicators that can aid in risk stratification. Additionally, this study assesses the effectiveness of different treatment strategies, including conservative management, minimally invasive interventions, and surgical approaches, in improving patient outcomes. By addressing these objectives, we aim to enhance clinical decision-making and optimise therapeutic protocols, reducing morbidity and mortality associated with this severe renal infection.

## Materials and methods

A retrospective observational study was conducted at Kasturba Hospital, Manipal, a tertiary care hospital in South India, from March 1, 2023, to September 30, 2023. The hospital records of the study period (2017-2022) were reviewed. A structured data collection process was followed to extract demographic, clinical, laboratory, radiological, and treatment details using the International Classification of Disease codes. Two independent researchers cross-verified the collected data to ensure accuracy and completeness.

The inclusion criteria used included patients aged >18 years, admitted with a diagnosis of EPN confirmed radiologically and requiring hospital admission during the period 2017 to 2022. The exclusion criteria used were age <18 years, patients with a suspected diagnosis of EPN who succumbed before confirmation of diagnosis, and patients with incomplete records. Demographic profiles along with signs and symptoms at presentation were documented from electronic medical records. Information regarding age, sex, and underlying medical conditions was obtained. Laboratory reports, including haematological, biochemical, and microbial culture reports, were collected. The laboratory variables included total leukocyte count (TLC), platelet count, sodium, potassium, hemoglobin A1c (HbA1c), serum urea, and creatinine (at admission, one month and three months), blood, urine culture, and sensitivity.

Radiological reports in the form of ultrasonography or computed tomography reports were collected. Severity of the EPN was noted as per Huang classification [7] in Table 1.

**Table 1:** Description of radiological severity of EPN Huang JJ, Tseng CC. Emphysematous pyelonephritis: clinicoradiological classification, management, prognosis, and pathogenesis. [7]

Class	Description
Class 1	Gas in collecting system only
Class 2	Parenchymal gas only
Class 3A	Extension of gas into perinephric space
Class 3B	Extension of gas into pararenal space
Class 4	EPN in solitary kidney, or bilateral disease

Treatment details in the form of the duration of antibiotics received, urological procedures like nephrostomy and pigtail drainage, DJ stenting, and nephrectomy if done were noted. Outcomes were documented in the form of death or discharge. Complications, including the occurrence of chronic kidney disease at three months follow-up were documented.

Clinical, biochemical, and microbiological data were recorded in a tabular form. Once the data was obtained, we decided to analyse the various laboratory variables to study the factors that could serve as a predictor of mortality in patients with EPN. Several risk factors associated with worse prognosis were analyzed to determine their role in the outcome of patients with EPN. Laboratory and haematological parameters like sodium, creatinine, TLC, platelet, neutrophil-to-lymphocyte (NL) ratio, HbA1c, and random blood sugar (RBS) were used to compare the survivor and mortality groups.

The study was approved by the Institutional Review Board and Ethics Committee of Kasturba Hospital, Manipal (Approval number: IEC2-365). Ethical guidelines were followed, ensuring patient confidentiality and anonymity. Due to the retrospective nature of the study, informed consent was waived by the ethics committee.

Definitions

Thrombocytopenia was defined as a platelet count less than 150,000/mL [8]. Hyponatremia was defined as serum Na <135 mEq/L [5]. Shock was defined as systolic pressure less than 90 mmHg [5]. Patients with an absolute increase in serum creatinine of ≥0.3 mg/dL after admission compared with baseline serum creatinine level were diagnosed with AKI [5]. Chronic kidney disease (CKD) was defined as high creatinine i.e. >1.4 mg/dl at three months [9]. 

Statistical methods

Statistical analysis was performed using SPSS 20.0 for Windows (IBM, Armonk, NY, USA). Qualitative data were expressed as percentages and frequencies. Quantitative data was analyzed using the independent samples t-test to compare continuous variables between the survivor and mortality groups. Results were expressed as mean ± standard deviation, and a p-value of <0.05 was considered statistically significant. This approach allowed for assessing differences in laboratory and clinical parameters to identify potential predictors of mortality in patients with EPN.

## Results

A total of 117 patients diagnosed with emphysematous pyelonephritis were included in the study. A total of 66 (56.4%) patients were female and 51 (43.6%) were males. The mean age of the study population was 55.5 years.

The most common symptom reported was flank pain, prevalent among 91 (77.8%) individuals. Fever followed closely, being experienced by 82 (70.1%) participants. Dysuria and hypotension were reported by 38 (32.5%) and 10 (8.5%) individuals, respectively. The most prevalent risk factor was DM, reported by 113 (96.6%) individuals, as mentioned below in Table 2.

**Table 2:** Demographic and clinical characteristics of patients with emphysematous pyelonephritis. Data are presented as absolute numbers (n) and percentages (%), where applicable, summarizing the total study population, gender distribution, mean age (years), primary symptoms at presentation, associated risk factors, radiological classifications, complications, and mortality. DKA - Diabetic Ketoacidosis AKI - Acute Kidney Injury CKD - Chronic Kidney Disease

Characteristic	Details	Number(n)	Percentage(%)
Total Patients		117	
Gender	Female	66	56.4%
	Male	51	43.6%
Mean Age		55.5	
Presenting Symptoms	Flank Pain	91	77.8%
	Fever	82	70.1%
	Dysuria	38	32.5%
	Hypotension	10	8.5%
Risk Factors	Diabetes Mellitus (DM)	113	96.6%
	Neutropenia	1	0.8%
	Renal Stones	39	33.3%
	Hydronephrosis	24	20.5%
Class	I	39	33.3%
	II	33	28.2%
	IIIa	11	9.4%
	IIIb	14	11.9%
	IV	8	6.8%
	Missing data	12	10.2%
Complications	DKA	4	3.4%
	AKI	53	45.3%
	AKI on CKD	9	7.7%
	CKD	32	27.3%
	Thrombocytopenia	30	25.6%
	Anemia	35	30%
	Septic Shock	7	6%
	Hydronephrosis	24	20.5%
Mortality		7	6%

In our study, blood and urine cultures were collected to identify positive results for various organisms as shown in Table 3. Out of the total samples analysed, the organism was isolated in 76 patients (64.9%). In 28 patients (23.9%), organisms were isolated in both blood and urine cultures. When considering the specific organisms isolated from both blood and urine cultures, E. coli was the most prevalent, with 64 cases (54.7%) followed by Klebsiella pneumoniae (n=7, 6%). Candida and methicillin-resistant Staphylococcus aureus (MRSA) were identified in two cases (1.7%) each, and Enterococcus faecalis was detected in one case (0.8%).

**Table 3:** Comprehensive summary of blood and urine culture results, antibiotic sensitivity patterns, treatment modalities, and urinary drainage interventions in the study population. Data include the total number of samples analyzed, frequency of organisms isolated, distribution of specific organisms, antibiotic regimens administered, average treatment duration, and interventions for urinary drainage, such as DJ stent placement, pigtail catheterization, and nephrostomy. Results are presented as absolute numbers (n) and percentages (%). ESBL - Extended-spectrum beta-lactamases

Characteristic	Details	Number (n)	Percentage (%)
Total Samples Analysed		117	100%
Organisms Isolated		76	64.9%
Isolated in Both Cultures	Blood and Urine	28	23.9%
Specific Organisms	E. coli	64	54.7%
	Klebsiella pneumoniae	7	6%
	Candida	2	1.7%
	MRSA	2	1.7%
	Enterococcus faecalis	1	0.8%
Antibiotic Sensitivity Pattern of E. coli	Total E. coli Cases	64	54.7%
	ESBL Positive	42	35.9%
	Pan-sensitive E. coli	8	6.8%
	Carbapenem-Resistant E. coli	1	0.8%
Average duration of treatment		17.25	
Antibiotics Used	Carbapenems (Meropenem, Imipenem)	50	42.7%
	3rd Generation Cephalosporins	32	27.4%
	2nd Generation Cephalosporins	12	10.3%
	Piperacillin-tazobactam	8	6.8%
	Fluoroquinolones	8	6.8%
	Colistin	1	0.8%
Interventions	DJ Stent Placement	65	55.6%
	Pigtail Catheter	8	6.8%
	Nephrostomy	1	0.8%

The antibiotic sensitivity pattern of E. coli was examined in our study. Among the cases of E. coli infection, 42 (35.9%) were found to be ESBL-positive. On the other hand, only a smaller subset of E. coli cases (n=8, 6.8%) showed pan sensitivity, meaning they were susceptible to a broad spectrum of antibiotics. Only one case (0.8%) showed carbapenem resistance.

The average duration of treatment was reported to be 17.25 days. The majority of the patients were treated with carbapenems like meropenem and imipenem (n=50), followed by third-generation cephalosporins like ceftriaxone and cefoperazone-sulbactum (n=32). Twelve patients were treated with cefuroxime, a second-generation cephalosporin. Eight patients each were treated with piperacillin-tazobactum and fluoroquinolones like ofloxacin respectively. One patient was treated with colistin. Among the patients, 65 individuals (55%) received a DJ stent, a medical device used to ensure proper urine drainage from the kidneys to the bladder. Additionally, a small number of patients underwent alternative interventions for urine drainage. Eight patients (6.8%) received a pigtail catheter, while one patient (0.8%) underwent nephrostomy, a procedure involving the placement of a tube directly into the kidney to drain urine. These treatment modalities, including antibiotic therapy and the use of DJ stents or alternative drainage methods, play crucial roles in managing the condition under study and promoting patient recovery.

**Figure 1:**
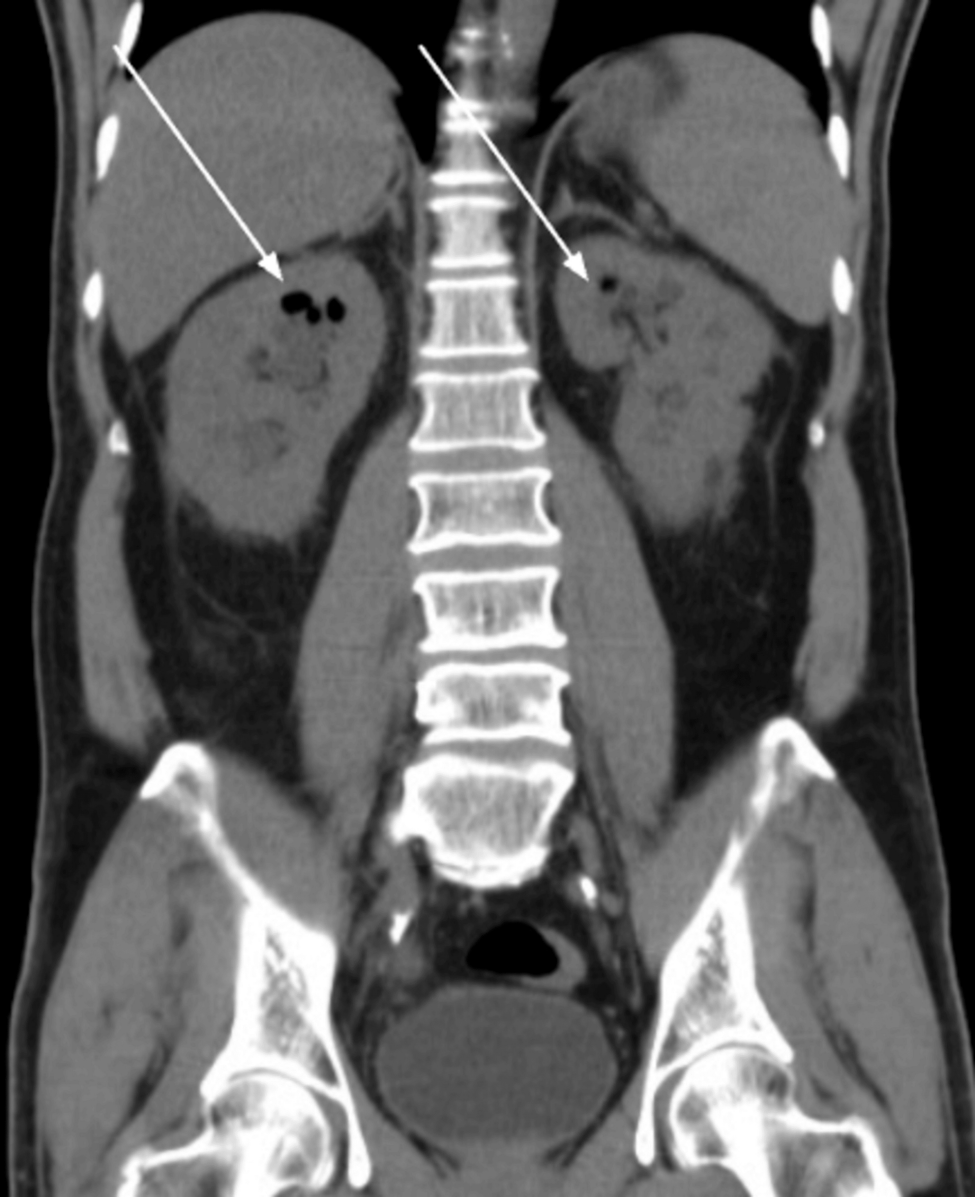
Image shows air filling in both kidneys suggestive of class 4 emphysematous pyelonephritis (EPN) (pointed by white arrows).

**Figure 2:**
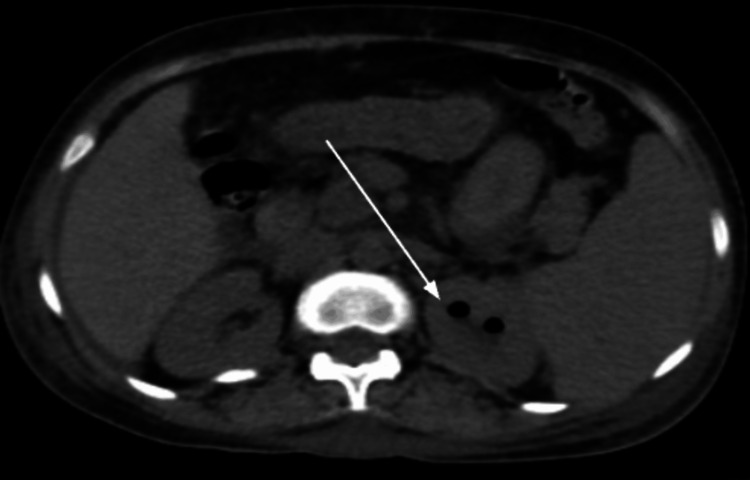
Image shows emphysematous changes in left kidney suggestive of Class 1 emphysematous pyelonephritis (EPN) (pointed by white arrows).

The classification of the participants in our study was based on a certain criterion as mentioned above. The 117 individuals were divided into radiological classes according to their imaging reports as shown in Figure 1 and Figure 2. The frequency distribution of the classes revealed that the majority of the participants (39 individuals, 33.3%) belonged to Class 1. Class 2 comprised 33 individuals (28.2%), representing the second highest proportion. Class 3a had 11 participants (9.4%), while Class 3b had 14 participants (11.9%). Finally, Class 4 had eight individuals (6.838%). It is important to note that 12 participants (10.2%) had missing data regarding their classification.

In our study, we identified several complications among the patients as mentioned in Table 2. Diabetic ketoacidosis (DKA) was reported in four (3.4%) individuals. AKI was observed in 53 (45.3%) participants, with an additional nine (7.7%) experiencing AKI on a background of CKD. Approximately 32 (27.3%) individuals had CKD. Thrombocytopenia affected 30 (25.6%) participants, while anaemia was seen in 35 (30%) individuals. Septic shock was reported in seven (6%) participants. Hydronephrosis was reported in 24 (20.5%) patients, and mortality was observed in seven (6%) individuals.

Several key laboratory variables were examined to assess risk factors predicting mortality using an independent samples t-test as mentioned in Table 4. These laboratory variables were included to study for differences between the survivor and mortality groups. Higher serum creatinine was significantly associated with mortality (p-value = 0.004).

**Table 4:** Comparison of key biochemical and haematological parameters between survivors and the mortality group. Data includes mean values with reference ranges for sodium, creatinine, total leukocyte count (TLC), platelet, neutrophil-to-lymphocyte (NL) ratio, HbA1c, and Random Blood Sugar (RBS). The results are presented as mean ± standard deviation (SD) for survivors and the mortality group. P-values were calculated using the t-test, with a significance threshold set at p < 0.05.

	Mean (Reference range)	Survivors	Mortality group	P value
Sodium (mmol/L)	129.1 (135-145)	129±6.2	130±6.1	0.71
Creatinine (mg/dL)	2.9 (0.7-1.2)	3.2±2.4	7.0±5.8	0.004
TLC (x 10^3 cells/µL)	14.6 (4-11)	14.7±7.6	12.2±8.2	0.51
Platelet (x 10^3 cells/µL)	228.0 (150-400)	233±136.6	130.7±32.7	0.13
NL Ratio	11.9 (1-2)	11.8±7.9	14.2±12.2	0.55
HbA1c (%)	14.2 (4-5.6)	14.4	10.3	0.86
RBS (mg/dl)	276.0 (70-140)	275	297	0.75

The average haemoglobin level was 10.220 g/dL (Normal: 13-16), while mean TLC was 14.626 x 10^3 cells/µL (Normal: 4-11 x 10^3), suggesting infectious aetiology. Average platelet count was 228.046 x 10^3 cells/µL (Normal: 150-400 x 10^3). The NL ratio, an important indicator of prognosis in sepsis, had an average value of 11.904 (Normal: 1-2). Mean sodium and potassium levels were 129.137 mmol/L (Normal: 135-145) and 4.729 mmol/L (Normal: 3.5-5), suggesting hyponatremia. The mean creatinine level at admission was 2.941 mg/dL (Normal: 0.7-1.2), and the mean RBS was 276.044 mg/dL (Normal: 70-140). Lastly, the average HbA1c level was 14.237% (Normal: 4-5.6). 

## Discussion

EPN is an uncommon, life-threatening renal infection that is characterised by the formation of intraparenchymal gas. The term 'EPN' was coined in 1962 by Schultz and Klorfein [10]. Women have been found to be affected more commonly by the infection as compared to men [11]. However, the ratio of female to male involvement varies. A study by Wan et al. found a 3:1 ratio of female/male predominance, whereas another study by Lu et al. found a 12:1 female dominance [12,5]. We found a ratio of 1.3, which is consistent with the current literature. The reason behind the gender disparity is believed to be due to a higher susceptibility to urinary tract infections in females [3]. 

Various factors like the presence of diabetes mellitus, urinary tract obstructions, immunosuppression, and structural abnormalities of the urinary tract have been found to be associated with EPN [13]. Diabetes (96.6%) and renal stones (33.3%) were the most common comorbidities of EPN in our cohort. While Huang et al. [7] found a prevalence of 96% diabetes and 22% urinary tract obstructions, Eswarappa et al. [13] reported a frequency of 98% diabetes and 7.8% renal calculi in their cohort of EPN patients. The higher predisposition in diabetes is mostly due to increased tissue glucose levels, which favour the growth of gas-producing bacteria and impair immune response [14]. In patients with EPN without diabetes, urinary albumin is believed to be acting as a substrate for the organism [15]. Flank pain and fever were the two most common initial presentations found in 77.8% and 70.1% of our patients, respectively. In their study, Huang et al. reported fever in 79% of their patients and flank pain in 71% representing the two most common symptoms [7].

E. coli is by far the most common causative organism isolated in EPN patients [5,7,16,17]. Our report also revealed E. coli (54.7%) as the most commonly isolated organism from the blood and urine cultures, followed by Klebsiella pneumoniae (6%). Candida was also isolated in two patients (1.7%) and has also been occasionally identified as a pathogen in EPN [4]. Yu-Chuan Lu et al. recommended third-generation cephalosporins as the first line of treatment in EPN caused by E. coli and Klebsiella, the two most common bacteria causing the infection [14]. However, with the increased and unregulated use of antibiotics, the rate of antibiotic resistance has increased significantly in the recent past, with 45% of patients found to be resistant to third-generation cephalosporins by Jain et al. [18]. Of the 64 (54.7%) E. coli-positive patients in our cohort, 42 (35.9%) were ESBL-positive. Keeping this in mind and while evaluating the antibiotic sensitivity patterns in our hospital, we initiated carbapenems as the first-line treatment in our patients.

While there are no specific signs and symptoms to diagnose EPN, poor response to antibiotics in diabetic patients with uncomplicated pyelonephritis should raise a suspicion to evaluate for EPN. A prompt CT scan of the abdomen should be taken to confirm the diagnosis and to plan treatment. Radiological classification of our patients was done based on the classification given by Huang and Tseng [7]. The majority of our patients were categorised as Class 1 (33.3%) and Class 2 (28.2%). A study by Elawdy et al. also revealed a similar distribution of EPN patients, with a majority falling under Class 1 and 2 [19]. It also reported favourable outcomes with medical management in patients with Class 1 and 2 EPN [19].

Interestingly, the management of EPN has evolved substantially in the last couple of decades. The treatment focus has changed over the years from radical nephrectomy to more conservative methods, such as antibiotics and percutaneous drainage techniques. This is due to the availability of effective antimicrobials and better imaging modalities facilitating easy drainage [16,20,21]. Invasive surgical approaches with early nephrectomy have shown poor outcomes with high mortality rates [22]. A study by Ahlering et al. revealed a high mortality rate of 42% in patients undergoing emergency nephrectomy [23]. The main advantages of minimally invasive treatments include sparing of nephrons, avoidance of major surgery in critically ill patients, and reduced need for renal support after recovery [16,21].

Improvements in the management strategies have helped improve the mortality rate significantly in the last two decades, with mortality ranging around 21% [4]. The success rate of conservative treatment described by Misgar et al. was 88.5% [4]. A recent meta-analysis of 1146 patients revealed a mortality rate of 10% in patients treated with minimally invasive techniques compared to a mortality rate of 26.5% in those treated with emergency nephrectomy [24]. In our study, a majority of the patients were managed with antibiotics (111, 94%). 55% of our patients underwent DJ stenting while 6.8% received a pigtail catheter for adequate urinary drainage. Only one patient (0.8%) underwent nephrostomy, a procedure involving the placement of a tube directly into the kidney to drain urine. Our study had a mortality rate of 6% (7 patients), which is lower than the 10% mortality rate reported by Desai et al. [24].

Wu et al. recommended a management algorithm for EPN in 2022 [3]. They suggested aggressive resuscitation in patients with EPN followed by initiation of broad spectrum antibiotics and emergent drainage to relieve urinary obstruction [3]. Carbapenems and third-generation cephalosporins are recommended as empirical antibiotics of choice depending on the local sensitivity patterns [3]. Typically a duration of at least two weeks of antibiotic therapy is recommended [3]. Failure to respond to antibiotics and progression of severity of infection may require nephrectomy in selected cases [3]. Follow-up CT scans and compliance with treatment are essential for achieving the best outcome in EPN patients.

Factors affecting mortality in patients with EPN are not completely known. A number of studies have investigated prognostic factors predicting mortality in EPN with varying conclusions. Huang et al. identified thrombocytopenia, altered consciousness, and perinephric extension as key mortality predictors [7], while Lu et al. emphasised the role of severe hypoalbuminemia (serum albumin <3 g/dl), polymicrobial infections, and dialysis dependency in predicting poor outcomes [5]. Shock, altered sensorium, and thrombocytopenia were associated with a higher risk of death in EPN patients in another study by Kangjam et al. [6]. These findings highlight the multifactorial nature of EPN prognosis, necessitating further evaluation of laboratory and clinical markers to refine risk stratification. Desai et al. in their meta-analysis suggested a similar increase in mortality in patients with altered sensorium, sepsis, shock, and thrombocytopenia [24]. However, they could not find an association between severe proteinuria and mortality [24]. Falagas et al. reported thrombocytopenia as a significant risk factor for mortality, but no association was found between diabetes status and mortality in EPN [25]. Shock (systolic BP <90 mmHg), disturbed consciousness, and raised serum creatinine (> 2.5 mg/dl) were associated with increased risk of death, but the results were based on only very limited data [25]. In our study, elevated serum creatinine at admission emerged as a significant predictor of mortality (p = 0.004). This aligns with findings from Falagas et al., which identified renal dysfunction as a key determinant of poor prognosis in EPN [25]. Elevated creatinine levels likely reflect severe renal compromise due to infection-induced ischaemia and septic shock, reinforcing the need for early nephron-sparing interventions in high-risk patients.

Interestingly, HbA1c was higher in the survivor group in our study and was not associated with higher mortality. This contradicts the conventional assumption that poor glycemic control worsens EPN outcomes. Lu et al. similarly reported no correlation between HbA1c and mortality, suggesting that acute infection severity may overshadow chronic glycemic status as a predictor of outcome [5]. Even in patients with poorly controlled diabetes (HbA1c >8%), there was no association seen between raised tissue glucose levels and increased risk of mortality or the need for dialysis, despite the possibility that these levels could create an environment that is conducive to the growth of gas-producing bacteria [13]. Further studies are warranted to elucidate the role of diabetes-related metabolic derangements in EPN prognosis. One possible explanation for these findings could be due to renal failure-induced alteration in HbA1c levels, which may not accurately reflect long-term glycemic status in critically ill patients.

Our study represents one of the largest EPN cohorts analysed to date. Most of our reported findings corroborate the previous clinical data. However, we found a strikingly low mortality rate of only 6% in our cohort, which is significantly lower than the historically reported rates of 20-40% in similar studies [4,24]. This difference may be attributed to improved diagnosis, the use of minimally invasive drainage techniques, and better critical and medical care. Additionally, the predominance of Class 1 and 2 EPN in our cohort (61.5%) may have contributed to the favourable survival outcomes.

Additionally, on comparing the survivor and mortality groups, we found that serum creatinine at admission was significantly associated with mortality. This emphasises the role of renal dysfunction in disease progression as shown in a previous study by Falagas et al. [25]. Also, this information can be used to raise a clinical suspicion while evaluating patients admitted with acute pyelonephritis, thereby avoiding the risk of missing a diagnosis of EPN. The low mortality rate observed in our cohort suggests that a combination of early diagnosis, aggressive resuscitation, and appropriate treatment may improve outcomes in EPN. Our findings reinforce the importance of using a combination of medical and minimally invasive drainage procedures to achieve the best outcome. Due to the unfavourable prognosis, clinicians should maintain a high index of suspicion for EPN in patients not responding to the routine management of pyelonephritis.

Limitations

This study has limitations inherent to all single-centre, retrospective studies with limited generalisability to broader populations. Data regarding the time to diagnosis from symptom onset and treatment initiation were not available, which may have impacted the assessment of disease progression and outcomes. Moreover, data regarding follow-up visits were not available for all the patients in the cohort due to the retrospective nature of the study. The relationship between radiological class, antibiotic type, and mortality could not be thoroughly evaluated due to variability in treatment approaches and incomplete data on antibiotic susceptibility patterns. Furthermore, the small number of non-diabetic patients in the study limited the ability to compare disease severity and outcomes between diabetic and non-diabetic groups. Future multicenter, prospective studies with standardised data collection are needed to validate these findings and provide more comprehensive insights into EPN management.

## Conclusions

EPN is an extremely dangerous renal infection with a high risk of mortality. However, early clinical suspicion, diagnosis, and treatment can lead to improved outcomes in patients. Women with diabetes represent a very susceptible population, while E. coli was the most frequently isolated pathogen. Radiological classification helps to stage the condition and guides appropriate treatment as well. The majority of the patients have early EPN (Class 1 and 2) and respond to a combination of antibiotics plus surgical drainage. We recommend early aggressive medical treatment and recommend that nephrectomy be considered only if patients deteriorate or do not improve on conservative treatment. Following a retrospective analysis, we identified elevated serum creatinine as a significant prognostic marker of mortality. This highlights the need for early renal function assessment in all suspected EPN cases. Our findings also underscore the importance of early diagnosis and aggressive supportive care. A low mortality in our study emphasises the importance of appropriate antimicrobial therapy guided by local resistance patterns and minimally invasive drainage techniques to improve survival in EPN patients.
